# Editorial: Monitoring and reducing errors in veterinary radiology

**DOI:** 10.3389/fvets.2026.1805635

**Published:** 2026-03-11

**Authors:** Sibylle Maria Kneissl

**Affiliations:** Department for Small Animals and Horses, University of Veterinary Medicine Vienna, Vienna, Austria

**Keywords:** artificial intelligence, cat, discrepancy, dog, error, horse, machine learning, veterinary diagnostic imaging

Diagnostic imaging stands at the center of modern veterinary medicine. It informs therapeutic decisions, guides surgical planning, and increasingly shapes outcome discussion. Yet, despite remarkable technological progress, diagnostic error remains an inherent and universal feature of radiologic practice. Errors may arise from perceptual or cognitive failure, methodological or technological constraints, or systemic pressures. Recognizing this reality is not a sign of weakness in the discipline—it is a necessary step toward strengthening it.

The Research Topic *Monitoring and reducing errors in veterinary radiology* was conceived to examine diagnostic error not as an isolated failure, but as a measurable and improvable component of clinical imaging. Across species, modalities, and analytical approaches, the contributions gathered here reflect a shared commitment: to move veterinary radiology toward greater transparency, reproducibility, and accountability.

A foundational element of this endeavor is reproducibility. Charles et al. evaluated intra- and interobserver agreement of the Pivot Point (PP) method for assessing carpal deformities in foals; the PP method applied equaled the median opinion of all observers. Their findings revealed good intraobserver reproducibility but limited agreement between readers—even when formal geometric measurements were applied. Notably, subjective visual assessment and structured PP evaluation showed substantial concordance within individual observers. This work challenges the assumption that increasing measurement complexity automatically reduces error. Instead, it highlights the importance of methodological clarity, standardized training, and—where possible—continuity of interpretation in longitudinal case management.

If reproducibility represents internal reliability, outcome correlation represents external validation. In comparing thoracic CT findings with surgical observations in dogs and cats, Brložnik et al. demonstrated high overall agreement, reinforcing the clinical value of CT in surgical planning. Yet discrepancies persisted, particularly in subtle lesions such as pulmonary bullae and radiolucent foreign bodies. By distinguishing perceptual errors from cognitive misinterpretations and true discrepancies, this study reframes error analysis as a diagnostic tool. Understanding *why* errors occur is a prerequisite to preventing them.

Interobserver variability in abdominal imaging was further explored by Kneissl et al., who demonstrated that even experienced clinicians may diverge in interpretation, particularly in anatomically complex or subtly abnormal regions. Rather than viewing disagreement as failure, such findings illuminate where methodological reporting and collaborative review may yield measurable improvements in consistency.

Artificial intelligence (AI) represents perhaps the most transformative development in this field. Ndiaye et al. compared board-certified veterinary radiologists with a widely used commercial AI system for canine and feline radiographs. The AI matched the performance of the best-performing radiologist in terms of overall accuracy and surpassed the performance of the median radiologist, particularly in cases of low ambiguity (AI: 0.962; median radiologist: 0.851). However, sensitivity for abnormal findings was lower, and no differential diagnoses were generated. Results of this study suggest that AI's most immediate strength lies in confirming normality and reducing variability, while human expertise remains indispensable in complex or ambiguous scenarios. The future of veterinary radiology will likely not be defined by replacement, but by integration—where complementary strengths are strategically aligned.

Technological innovation is further exemplified by the development of a deep learning model for automatic detection of narrowed intervertebral disc spaces in canine radiographs by Park et al. The model achieved substantial agreement with clinicians while dramatically reducing interpretation time (0.104 s per image). However, acute non-compressive nucleus pulposus extrusion (ANNPE) was not detected by the AI model because ANNPE does not necessarily cause radiographically visible intervertebral disc space narrowing and therefore falls outside the model's diagnostic scope. This highlights a critical insight: although automation enhances reproducibility, it does not eliminate the need for multimodal clinical reasoning, which draws on multiple sources of information. The final assessment, including quantification, must remain embedded within a comprehensive approach that combines a neurological examination, radiographs, and MR imaging.

Finally, Argue et al. examined the inter-observer agreement for orthopedic lesions in pre-sale thoroughbred yearling radiographs. Increasing interpretative ambiguity (case difficulty) reduces interobserver agreement and diagnostic stability. Lesions characterized by subtle morphologic change demonstrate greater variability in detection and risk classification, underscoring the potential value of standardized grading frameworks and AI-assisted triage in complex or borderline cases. The complementary error profiles of human observers and AI systems suggest that optimal diagnostic performance may emerge from layered interpretation models, i.e., layer 1: AI-screening for rapid triage or standardized quantification; layer 2: human applies contextual reasoning by integrating patient history or clinical signs at the time of presentation. Rather than seeking to eliminate variability entirely, error reduction may be achieved by strategically balancing human sensitivity with algorithmic specificity.

Collectively, the six contributions reveal that reducing diagnostic error requires more than technical refinement. Although a methodological approach improves consistency, it cannot fully eliminate interobserver variability. Outcome validation strengthens accountability but depends on systematic follow-up. AI increases efficiency and specificity but must be integrated thoughtfully to avoid new forms of bias. Traditionally, diagnostic ambiguity was viewed as an undesirable source of variability. However, it can be reframed as measurable information. By quantifying disagreement between observers or uncertainty in AI predictions, ambiguity itself becomes an objective signal that a case warrants closer review. Rather than eliminating uncertainty, modern systems can use it strategically to guide layered interpretation and targeted oversight.

Perhaps most importantly, this Research Topic signals a cultural evolution. Monitoring errors must transition from a reactive exercise to a proactive framework for continuous improvement. Open discussion of discrepancies, structured reporting systems, education in cognitive bias, peer consultation, and responsible adoption of emerging technologies together form the architecture of a more resilient diagnostic discipline.

In conclusion, *Monitoring and Reducing Errors in Veterinary Radiology* offers more than a collection of studies—it outlines a pathway. Through methodological rigor, technological innovation, and professional transparency, veterinary radiology can continue to evolve toward increased diagnostic reliability, improved patient outcomes, and sustained trust in imaging as a cornerstone of clinical decision-making.

